# Enhanced cognitive behaviour therapy for adults with anorexia nervosa: A UK–Italy study

**DOI:** 10.1016/j.brat.2012.09.010

**Published:** 2013-01

**Authors:** Christopher G. Fairburn, Zafra Cooper, Helen A. Doll, Marianne E. O'Connor, Robert L. Palmer, Riccardo Dalle Grave

**Affiliations:** a Oxford University Department of Psychiatry, Warneford Hospital, Oxford OX3 7JX, UK; b Norwich Medical School, University of East Anglia, Norwich NR4 7TJ, UK; c Department of Health Sciences, Leicester University, 22-28 Princess Road West, Leicester LE1 6TP, UK; d Villa Garda Hospital, Department of Eating and Weight Disorder, I-37016 Garda, VR, Italy

**Keywords:** Anorexia nervosa, Treatment, Cognitive behaviour therapy, Eating disorder

## Abstract

Anorexia nervosa is difficult to treat and no treatment is supported by robust evidence. As it is uncommon, it has been recommended that new treatments should undergo extensive preliminary testing before being evaluated in randomized controlled trials. The aim of the present study was to establish the immediate and longer-term outcome following “enhanced” cognitive behaviour therapy (CBT-E). Ninety-nine adult patients with marked anorexia nervosa (body mass index ≤ 17.5) were recruited from consecutive referrals to clinics in the UK and Italy. Each was offered 40 sessions of CBT-E over 40 weeks with no concurrent treatment. Sixty-four percent of the patients were able to complete this outpatient treatment and in these patients there was a substantial increase in weight (7.47 kg, SD 4.93) and BMI (2.77, SD 1.81). Eating disorder features also improved markedly. Over the 60-week follow-up period there was little deterioration despite minimal additional treatment. These findings provide strong preliminary support for this use of CBT-E and justify its further evaluation in randomized controlled trials. As CBT-E has already been established as a treatment for bulimia nervosa and eating disorder not otherwise specified, the findings also confirm that CBT-E is transdiagnostic in its scope.

## Highlights

► CBT-E is designed to address eating disorder psychopathology. ► The findings show that CBT-E can be used with patients with marked anorexia nervosa. ► Two-thirds of the patients completed treatment. ► Among the completers there was a substantial, and well maintained, response. ► The findings confirm that CBT-E is transdiagnostic in its scope.

## Introduction

Anorexia nervosa in adulthood has been described as “one of the most difficult psychiatric disorders to treat” (Halmi et al., 2005). Reluctance to engage in treatment is common, and in those who do accept treatment the outcome is often poor. Hospitalization is essential in some cases and it generally results in weight gain, but it is expensive and disruptive and often followed by weight loss (Carter et al., 2009; Kaplan et al., 2009; Walsh et al., 2006). A treatment that produced enduring change would be of great value, especially if it were deliverable on an outpatient basis (Bulik, Berkman, Brownley, Sedway, & Lohr, 2007).

Anorexia nervosa is also difficult to study (Agras et al., 2004; Bulik et al., 2007; Fairburn, 2005; Halmi, 2008; Lock et al., 2012). This is because of its relative rarity, the associated medical risks, the lengthy duration of treatment, and the importance of follow-up to determine whether treatment effects persist over time. Nine studies of psychosocial treatments have been published (Bulik et al., 2007) and several have run into major difficulties (Halmi et al., 2005; Lock et al., 2012). Almost all the studies have been small in size, the average number of patients per condition being less than 20. These methodological challenges, together with the disappointing or inconclusive results of the studies to date, have led to the suggestion that new treatments for anorexia nervosa should undergo extensive preliminary testing before being considered eligible for evaluation in randomized controlled trials (Agras et al., 2004; Fairburn, 2005; Halmi et al., 2005; Lock et al., 2012). Alternatively it has been proposed that the focus of research should shift away from adults and on to the treatment of adolescents as they appear to be easier both to treat and to study (Halmi, 2008).

Cognitive behaviour therapy is a potential candidate as an outpatient treatment for anorexia nervosa since it is the leading empirically supported treatment for bulimia nervosa (National Institute for Clinical Excellence, 2004; Shapiro et al., 2007), a disorder with overlapping psychopathology. The cognitive behavioural treatment for bulimia nervosa has recently been adapted with the goal of making it suitable for any form of eating disorder, including anorexia nervosa (Fairburn, 2008; Fairburn, Cooper, & Shafran, 2003). To this end, the new “enhanced” form of the treatment (CBT-E) focuses on modifying the mechanisms thought to perpetuate all forms of eating disorder psychopathology (Fairburn et al., 2003). The treatment has been shown in two independent studies (combined *N* = 245) to produce sustained change in those eating disorder patients who are not significantly underweight, whatever their DSM diagnosis (Byrne, Fursland, Allen, & Watson, 2011; Fairburn et al., 2009). The utility of the treatment with the remaining eating disorder patients, those with anorexia nervosa, has yet to be established.

In light of the recommendation that new treatments for adults with anorexia nervosa undergo extensive preliminary evaluation, we studied the effects of CBT-E in two representative, markedly affected, patient samples. Many of these patients would ordinarily have been hospitalised. We chose to include patients who were significantly underweight in order to test the full potential of the new treatment.

The study was designed to address four key clinical questions. First, among adults with marked anorexia nervosa, what proportion is able to complete this outpatient treatment? Second, among those patients who can complete the treatment, what is their outcome? Third, are the changes sustained? And fourth, are there baseline variables that predict treatment completion? By studying two independent patient samples we were also able to determine whether there is consistency in these patients' response to CBT-E.

## Method

### Design

Two samples of patients were recruited, one from the UK and the other from Italy. Both comprised patients with anorexia nervosa who had a body mass index (BMI; weight in kg/height^2^ in m) of 17.5 or below, a commonly used threshold for anorexia nervosa. All the patients were offered CBT-E and, if they accepted, were provided with 40 sessions of treatment over 40 weeks. This was the only psychological or behavioural intervention that they received. The patients were then entered into a closed follow-up period lasting 60 weeks during which they received no further treatment unless it was judged necessary on clinical grounds. The studies were approved by the local human subjects committees.

### Setting and participants

The UK sample was recruited from consecutive referrals by family doctors and other clinicians to two well-established National Health Service eating disorder clinics, one serving central Oxfordshire and the other serving Leicestershire. Each referral was assessed by a senior clinician who established the patient's diagnosis and eligibility for the study. The Italian sample was recruited in a similar way. It comprised consecutive referrals to an eating disorder clinic serving the Verona area.

The UK patients had to fulfil the DSM-IV diagnostic criteria for anorexia nervosa (American Psychiatric Association, 1994), bar the amenorrhoea criterion, and to have a BMI between 15.0 and 17.5. In addition, they had to be aged between 18 and 65 years and provide written informed consent after receiving a complete description of the study. The entry criteria for the Italian sample were the same except that there was no lower BMI limit. The exclusion criteria for both samples were as follows: i) the patient being unsafe to manage on an outpatient basis (*N* = 4 and 0; UK and Italian samples respectively); ii) having received in the previous year a specialist treatment for anorexia nervosa (*N* = 4 and 0); iii) having a co-existing Axis 1 psychiatric disorder that precluded immediate eating disorder-focused treatment, such as psychosis or drug dependence (*N* = 11 and 4); and iv) not being available for the 40 week period of treatment (*N* = 4 and 4). If it was thought that there was a comorbid major depressive disorder in addition to the eating disorder, this was treated with antidepressant medication prior to starting CBT-E. Patients who were already receiving psychotropic medication were weaned off this prior to entering the study (*N* = 2 and 2), an exception being clinically warranted antidepressant medication (*N* = 22 and 17) which was kept stable during treatment with CBT-E.

**Fig. 1:**
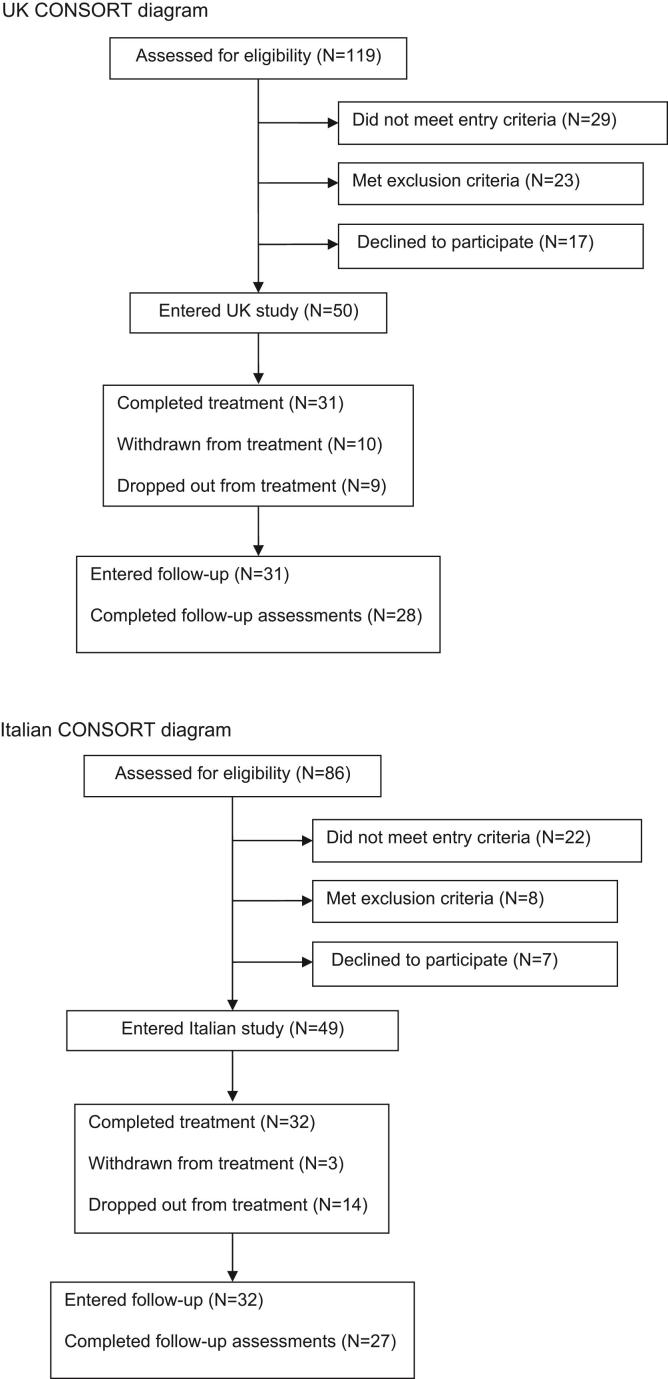
CONSORT diagrams for the UK and Italian samples.

Patients who met the study entry criteria at the initial assessment were offered two further appointments in order to describe the treatment and obtain consent. Fig. 1 shows the recruitment and retention figures for the UK and Italian participants.

### Intervention

CBT-E is a treatment for patients with eating disorder psychopathology. With patients who are underweight, it has three phases. In the first, the emphasis is on increasing patients' motivation to change. Then, if willing, patients are helped to regain weight while at the same time they tackle their eating disorder psychopathology including their extreme concerns about shape and weight. In the final phase the emphasis is on helping them develop personalized strategies for identifying and immediately correcting any setbacks. The focused version of CBT-E was used as it appears to have greater clinical utility than the broad version (Fairburn et al., 2009). A detailed manual for therapists is available (Fairburn, Cooper, Shafran et al., 2008).

Treatment involved 40, 50-min, one-to-one sessions over 40 weeks. A single therapist treated each patient. There were seven UK therapists of whom five were clinical psychologists and two were psychiatric nurse specialists. The Italian site had four therapists, all of whom were clinical psychologists. All the therapists had prior generic clinical experience and experience treating patients with eating disorders, and each received six months initial training from CGF and ZC (UK) and RDG and CGF (Italy). Weekly supervision meetings were held throughout the study and were led by CGF and ZC (UK) and RDG (Italy). The therapists had six-monthly booster workshops led by CGF. All the sessions were recorded and these recordings were used as part of supervision to ensure that the treatment was well implemented.

### Assessment

The assessment points were before treatment, at the end of treatment and 60 weeks later.

#### Body weight and body mass index

Weight was measured using a beam balance scale and height was measured using a wall-mounted stadiometer.

#### Eating disorder features

The UK patients were assessed using the 16th edition of the Eating Disorder Examination interview (Fairburn, Cooper, & O'Connor, 2008) (EDE) and its self-report version (Fairburn & Beglin, 2008) (EDE-Q6.0). The assessors had no involvement with treatment. The Italian patients were assessed using the Italian translation of the EDE-Q6.0.

#### General psychiatric features

In the UK sample, these were measured using the Brief Symptom Inventory (Derogatis & Spencer, 1982) (BSI), a short version of the Symptom Checklist-90 (Derogatis, 1977) (SCL). In the Italian sample the full SCL was used. The two instruments generate the same Global Severity Index (GSI).

### Statistical analysis

The statistical analysis was undertaken by HAD using standard treatment research data analytic procedures. Data are presented as *N* (%) for categorical data and as means (with standard deviation, SD) or medians (with range) for continuous data. Differences between groups were expressed as difference in proportion or relative risk (RR) for categorical data and as mean difference for continuous data. Chi-squared (*χ*^2^) or Fisher's exact tests (as appropriate) were used to compare categorical measures between the two groups, and *t*-tests or Mann–Whitney tests (as appropriate for the distribution of the data) to compare continuous measures. McNemar tests for categorical data and paired *t*-tests or Wilcoxon matched pairs signed rank test (as appropriate) for continuous data were used to compare differences within groups. Logistic regression analyses were used to identify independent predictors of outcome in terms of treatment completion at follow-up. Statistical significance was taken at two-sided *p* < 0.05 throughout, with 95% confidence intervals (CI) used to express the uncertainty in the data.

## Results

### The two samples

Recruitment continued until 99 patients had entered the study (UK, *N* = 50; Italy, *N* = 49). The demographic characteristics of the two samples are shown in Table 1. They primarily consisted of single, female patients in their mid-20s. The eating disorder was well established in most cases with the mean duration of anorexia nervosa being three years. Table 2 shows their clinical characteristics. Both samples were substantially underweight, the Italian sample weighing significantly less than the UK one (*p* < 0.001) reflecting the difference in the BMI entry criteria at the two sites. In most other respects the UK sample had significantly higher levels of psychopathology than the Italian one.

### Intent-to-treat findings at end of treatment and 60-week follow-up

Although the primary goal of this study was to determine the proportion of patients with marked anorexia nervosa who can complete this outpatient treatment, and their treatment response, intent-to-treat data are reported in Table 2. The method of data imputation involved moving the last available data point forward as this has been most commonly used approach in the studies to date. It can be seen that there was a marked increase in weight. By the end of treatment the mean BMI had increased from 16.1 (SD 1.2) to 17.9 (SD 1.8) and over the 60-week period of follow-up it remained stable (mean BMI 17.8, SD 2.0). The increase in weight was accompanied by a decrease in eating disorder psychopathology and general psychiatric features.

### Question 1 – What proportion of patients complete CBT-E?

Two-thirds of the patients in UK and Italian samples (63/99, 63.6%) completed the full 40 weeks of treatment (UK sample, *n* = 31, 62%; Italian sample *n* = 32, 65%; difference 3.3%, 95% CI 16–22%, *χ*^2^ *p* = 0.89). There were two inter-related reasons for non-completion. The first was being withdrawn on clinical grounds, either due to concerns about the patient's physical health or because of sustained lack of progress (UK sample *n* = 10, 20%; Italian sample *n* = 3, 6%; RR 3.25 (95% CI 0.96–11.2); *χ*^2^ *p* = 0.08). The second reason was ceasing to attend (UK sample *n* = 9, 18%; Italian sample *n* = 14, 29%; RR 0.63, 95% CI 0.30–1.32, *χ*^2^ *p* = 0.32). The patients who were withdrawn were highly symptomatic when they left the study and, without exception, they were referred for more intensive treatment.

### Question 2 – What is the outcome among those who complete CBT-E?

There was a substantial response to CBT-E among the treatment completers which was similar in the UK and Italian samples (see Table 3). The mean weight gain was 7.47 kg (SD 4.93; 95% CI 6.23–8.71; *p* < 0.001), equivalent to a BMI increase of 2.77 (SD 1.81; 95% CI 2.32–3.23). Over sixty percent (62%, 39/63) achieved a BMI ≥ 18.5. Eating disorder psychopathology and general psychiatric features also improved substantially with the mean global EDE-Q score among treatment completers decreasing by 1.89 (SD 1.46; 95% CI 1.52–2.26; *p* < 0.001) and mean GSI decreasing by 0.77 (SD = 0.58; 95% CI 0.63–0.92; *p* < 0.001). Almost 90 percent (88%, 55/63) had minimal residual eating disorder psychopathology, defined as having a global EDE-Q score below 1 SD above the community mean (Mond, Hay, Rodgers, & Owen, 2006) (i.e., <2.77).

### Question 3 – Are the changes sustained following CBT-E?

There was high compliance with follow-up with 84% (53/63) of the treatment completers being successfully reassessed. A minority of the 63 patients required further treatment during the 60 weeks of follow-up: three UK and two Italian patients received significant additional treatment, and four UK and three Italian patients were given one to five brief CBT-E “booster” sessions.

**Fig. 2:**
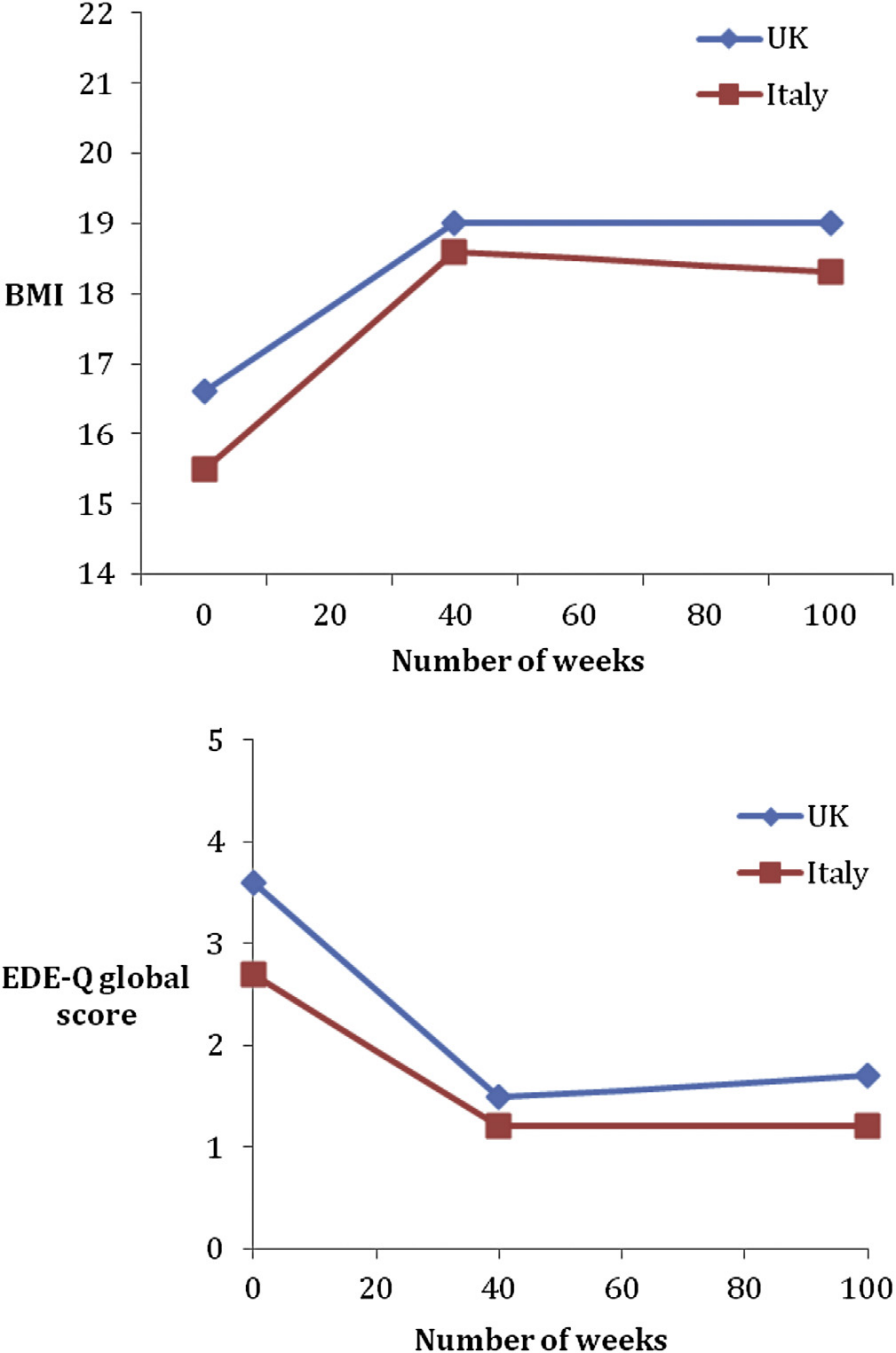
Mean body mass index (BMI) and eating disorder psychopathology (global EDE-Q) over 40 weeks of treatment and 60 weeks of follow-up among those patients who completed CBT-E (*n* = 63).

Overall the changes made during treatment were well maintained (see Table 3). There was a slight deterioration in mean weight and BMI, and the same was true of eating disorder features and general psychiatric symptoms (see Fig. 2). As a result, the proportion with a BMI ≥ 18.5 fell from 62% (39/63) to 55% (30/55). Similarly, the proportion with minimal residual eating disorder psychopathology deceased from 87% (55/63) to 78% (43/55).

### Question 4 – Are there baseline predictors of treatment completion?

There were no statistically significant relationships between study site, age, eating disorder duration or BMI and whether or not the patient completed treatment. Treatment completion was, however, significantly associated with severity of eating disorder and general psychopathology with those with greater psychopathology being less likely to complete. Global EDE-Q, EDE-Q shape and weight concern subscales, GSI score, and the frequency of binge eating and purging were significantly higher in non-completers than completers (at least *p* < 0.05), with the strongest relationships observed in terms of global EDE-Q and EDE-Q weight concern scores and the presence and frequency of purging (all *p* < 0.01). On multiple regression, only EDE-Q weight concern score and purging retained an independent effect, with the adjusted RRs for treatment completion for patients with EDE-Q weight concern score ≥ 3 being 0.29 (95% CI 0.11–0.79) and that for the presence of purging being 0.36 (0.14–0.91).

## Discussion

The aim of this study was to obtain robust data on the outcome following a new outpatient treatment for anorexia nervosa, a notoriously treatment-resistant condition when present in adults (Attia & Walsh, 2009; Halmi et al., 2005; Walsh et al., 2006). To achieve this aim, two independent and sizeable cohorts of patients were treated with CBT-E, and then entered into a 60-week closed period of follow-up. All the patients at recruitment had a BMI of 17.5 or below.

There were three main findings. The first was that two-thirds of the patients in both samples were able to complete this lengthy treatment. The remaining one third either ceased to attend or was withdrawn due to concerns about their physical health or lack of progress. The great majority of these patients were highly symptomatic at the point that treatment stopped. The fact that a third of the patients did not complete treatment is not surprising given that over half started treatment with a BMI below 16.5, a threshold recently recommended for hospitalization (Attia & Walsh, 2009) (UK sample *n* = 22, 44%; Italian sample *n* = 31, 63%).

The second finding was that among those who completed CBT-E there were substantial improvements in weight and eating disorder psychopathology. This was true of both samples. The mean weight gain was 7.5 kg (16.5 lb) with over sixty percent of the patients gaining sufficient weight to enter the WHO's healthy BMI range. In addition, almost ninety percent had minimal eating disorder psychopathology at the end of treatment, despite the weight gain. This is of note as in anorexia nervosa concerns about eating, shape and weight are aggravated by eating more, gaining weight and changing shape, and they are likely to contribute to the high relapse rate that is typically seen.

The third finding is therefore of particular interest. It concerns the stability of the changes obtained. Despite there being little exposure to further treatment, the changes were generally well maintained with there being only a slight deterioration in weight and eating disorder features. This is in marked contrast to the reports of high rates of relapse over the 12 months following hospitalization, even with ongoing therapeutic input (Carter et al., 2009; Kaplan et al., 2009; Walsh et al., 2006).

The intent-to-treat analyses were included to allow comparisons to be made with other studies, although doing so is complicated by the fact that patient samples differ in their characteristics and those to date have been markedly smaller than the present one. Two of the key outcome variables can be compared across the various studies. The first is completing treatment. Two-thirds of the present study's patients completed CBT-E, a completion rate that is typical for this type of patient group (Dare, Eisler, Russell, Treasure, & Dodge, 2001; McIntosh et al., 2005). The second variable is weight gain. The mean intent-to-treat weight gain was 5.0 kg. This compares favourably with the mean weight gain of 2.7 kg achieved by the only other outpatient study to report weight change in similarly underweight patients (Dare et al., 2001). It also compares well with the weight gains obtained with a generic form of CBT, interpersonal psychotherapy and a form of specialist clinical management in a less severely affected patient group (McIntosh et al., 2005).

The present study had certain strengths. First, the recruitment of two parallel samples, both large for studies of the treatment of anorexia nervosa, allowed us to determine whether the findings are likely to be robust. Second, the patients were recruited from consecutive referrals to long-established eating disorder clinics, each providing the main clinical service for their local area. The findings are therefore likely to be generalizable to mainstream clinical services elsewhere. Third, as noted earlier, the cases were not mild. Fourth, in contrast with many other studies, the index treatment, CBT-E, was these patients' sole psychological treatment: no other interventions were taking place in the background. Lastly, the patients were followed up for over a year after completing CBT-E, the period when relapse is most likely to occur.

Given its specific aims, the study had three main limitations. The first is that the findings cannot be generalized to patients with a BMI below 15.0 or above 17.5. Second, a longer period of follow-up would have been desirable to determine the stability of the changes in the long term. Third, while the samples were large for this comparatively uncommon disorder, the study only had modest statistical power for detecting site effects. Larger multisite studies would be needed to confirm that the effects of CBT-E are replicable. If a broader perspective on the study is taken, the other main limitation is that CBT-E was not compared with another treatment. This was intentional and in line with the recommendations outlined earlier. It does mean that no conclusions can be drawn regarding the effectiveness of CBT-E versus other approaches.

What the present study provides is robust benchmark data on the immediate and longer-term outcome following the use of CBT-E to treat adults with anorexia nervosa. Two-thirds of the patients were able to complete the treatment and among them there were substantial improvements in weight and eating disorder features that were well maintained. These findings are sufficiently promising to justify the evaluation of CBT-E in randomized controlled trials. The findings also confirm that CBT-E is a “transdiagnostic” treatment as there are now data supporting its use in anorexia nervosa, bulimia nervosa and eating disorder not otherwise specified. It can also be used with adolescents (Dalle Grave, Calugi, Doll, & Fairburn, 2013) and with inpatients (Dalle Grave, Calugi, Conti, Doll, & Fairburn, 2012).

## Funding

The study was funded by two programme grants from the Wellcome Trust, London (046386). CGF is supported by a Principal Research Fellowship from the Wellcome Trust (046386). The Wellcome Trust had no involvement in the design or execution of the study.

## Figures and Tables

**Table 1:** Characteristics of the two samples at baseline. Data are shown as mean (SD) unless otherwise indicated.

	UK sample (*N* = 50)	Italian sample (*N* = 49)	Total sample (*N* = 99)	*p*-Value
Age, years	23.4 (5.8)	24.6 (5.2)	24.0 (5.5)	*t*-Test *p* = 0.30
Gender, *n* (%) female	48 (96.0%)	48 (98.0%)	96 (97.0%)	Fisher's *p* = 1.00

Ethnicity, *n* (%)				
White	45 (90.0%)	49 (100.0%)	94 (94.9%)	*χ*^2^*p* = 0.076
Asian	3 (6.0%)	0	3 (3.0%)	
Mixed	2 (4.0%)	0	2 (2.0%)	

Marital status, *n* (%)				
Single, never married	43 (86.0%)	42 (89.4%)	85 (87.6%)	*χ*^2^*p* = 0.50
Married or living as such	5 (10.0%)	2 (4.3%)	7 (7.2%)	
Separated or divorced	2 (4.0%)	3 (6.4%)	5 (5.2%)	

Occupation, *n* (%)				
Full-time employment	15 (30.0%)	20 (40.1%)	35 (35.4%)	*χ*^2^*p* = 0.13
Student	32 (64.0%)	22 (44.9%)	54 (54.5%)	
Homeworker	1 (2.0%)	5 (10.2%)	6 (6.1%)	
Unemployed	2 (4.0%)	2 (4.1%)	4 (4.0%)	

Duration of eating disorder, years, median (range)	3.0 (0–24.0)	3.0 (0–17.0)	3.0 (0–24.0)	M-W *p* = 0.41

**Table 2:** Characteristics of the two samples before treatment, after treatment and at 60-week follow-up. Intent-to-treat data shown as mean (SD) unless otherwise stated.

	Before treatment			After treatment			At 60-week follow-up		
	UK (*N* = 50)	Italy (*N* = 49)	Total (*N* = 99)	UK (*N* = 50)	Italy (*N* = 49)	Total (*N* = 99)	UK (*N* = 50)	Italy (*N* = 49)	Total (*N* = 99)
Body weight (kg)	46.4 (4.5)	41.6 (6.0)***	43.9 (5.7)	50.9 (7.4)	46.9 (6.2)	48.9 (7.1)***	51.1 (7.7)	46.3 (6.8)	48.7 (7.6)***

Body mass index (kg/m^2^)	16.5 (0.7)	15.7 (1.4)***	16.1 (1.2)	18.1 (1.9)	17.7 (1.8)**	17.9 (1.8)***	18.2 (2.0)	17.5 (1.9)**	17.8 (2.0)***
BMI ≥ 18.5, *n* (%)	0	0	0	24 (48.0%)	17 (34.7%)	41 (41.4%)***	22 (44.0%)	12 (24.5%)	34 (34.3%)***

Eating disorder psychopathology									
Overall severity (global EDE)	3.33 (1.3)	–	3.33 (1.3)	2.30 (1.7)	–	2.30 (1.7)***	2.41 (1.7)	–	2.41 (1.7)***
Overall severity (global EDE-Q)	3.98 (1.4)	2.87 (1.5)***	3.44 (1.6)	2.35 (1.7)	1.81 (1.7)	2.09 (1.7)***	2.49 (1.7)	1.78 (1.7)*	2.13 (1.73)***
Global EDE-Q < 1 SD above the community mean,^a^*n* (%)	10 (20.0%)	22 (46.8%) **	32 (33.0%)	32 (64.0%)	34 (72.3%)	66 (68.0%)***	29 (58.0%)	33 (70.2%)	62 (63.9%)***
Dietary restraint (EDE-Q subscale)	3.83 (1.9)	3.08 (2.0)	3.46 (2.0)	2.12 (1.8)	1.67 (2.1)	1.90 (1.96)***	2.30 (1.8)	1.81 (2.1)**	2.06 (1.97)***
Eating concern (EDE-Q subscale)	3.76 (1.3)	2.68 (1.6)**	3.24 (1.6)	2.08 (1.7)	1.55 (1.5)	1.82 (1.65)***	2.32 (1.8)	1.49 (1.6)**	1.92 (1.74)***
Shape concern (EDE-Q subscale)	4.31 (1.6)	3.00 (1.7)***	3.68 (1.8)	2.92 (1.9)	2.18 (1.9)*	2.56 (1.97)***	2.54 (2.0)	2.03 (1.8)	2.29 (1.92)***
Weight concern (EDE-Q subscale)	4.01 (1.7)	2.69 (1.5)***	3.37 (1.8)	2.18 (1.7)	1.67 (1.7)	1.93 (1.75)***	2.69 (1.6)	1.58 (1.6)	2.15 (1.73)***

Eating disorder behaviour (EDE-Q)									
Objective bulimic episodes, *n* (%) present	23 (46.9%)	12 (24.5%)*	35 (35.7%)	19 (38.0%)	9 (18.4%)*	28 (28.3%)	19 (38.0%)	9 (18.4%)*	28 (28.3%)
If present, episodes/28 days, median (range)	3.0 (1–42)	4.0 (1–40)	3.0 (1–42)	3.0 (1–42)	4.0 (1–28)	3.0 (1–42)	3.0 (1–42)	12.0 (2–28)	3.0 (1–42)
Self-induced vomiting, *n* (%) present	11 (22.0%)	16 (32.7%)	27 (27.3%)	11 (22.0%)	8 (16.3%)	19 (19.2%)	12 (24.0%)	11 (22.4%)	23 (23.2%)
If present, episodes/28 days, median (range)	10.0 (1–45)	9.5 (1–60)	10.0 (1–60)	3.0 (1–42)	9.5 (1–60)	4.0 (1–60)	3.0 (1–42)	10.0 (1–60)	4.0 (1–60)
Laxative misuse, *n* (%) present	8 (16.0%)	8 (16.3%)	16 (16.2%)	8 (16.0%)	5 (10.2%)	13 (13.1%)	9 (18.0%)	7 (14.3%)	16 (16.2%)
If present, episodes/28 days, median (range)	12.0 (2–47)	11.5 (3–84)	12.0 (2–84)	2.0 (1–6)	30.0 (3–84)	3.0 (1–84)	2.0 (1–20)	10.0 (3–84)	3.0 (1–84)

General psychiatric features, GSI	1.80 (0.8)	1.36 (0.7)**	1.59 (0.8)	1.27 (1.0)	0.91 (0.7)	1.09 (0.9)	1.36 (0.9)	0.80 (0.6)**	1.09 (0.8)

EDE – Eating Disorder Examination (version 16.0D) (Fairburn, Cooper, & O'Connor 2008). EDE-Q – Eating Disorder Examination Questionnaire (version 6.0) (Fairburn & Beglin, 2008). GSI – Global Severity Index (Derogatis, 1977; Derogatis & Spencer, 1982). *p < 0.05; **p < 0.01; ***p < 0.001 UK vs Italian patients and total sample vs baseline.

a Global EDE-Q less than 1SD above community EDE-Q mean for young adult women (Mond et al., 2006) (i.e., <2.77).

**Table 3:** Characteristics of the two samples before treatment, after treatment and at 60-week follow-up among those who completed treatment. Data are shown as mean (SD) unless otherwise stated.

	Before treatment			After treatment			60-Week follow-up		
	UK (*N* = 31)	Italy (*N* = 32)	Total (*N* = 63)	UK (*N* = 31)	Italy (*N* = 32)	Total (*N* = 63)	UK (*N* = 28)	Italy (*N* = 27)	Total (*N* = 63)
Body weight (kg)	46.8 (4.2)	40.7 (5.5)***	43.7 (5.7)	53.5 (6.4)	48.9 (5.1)**	51.2 (6.1)***	53.3 (5.7)	47.4 (6.2)**	50.4 (6.6)***

Body mass index (kg/m^2^)	16.6 (0.6)	15.5 (1.4)***	16.0 (1.2)	19.0 (1.4)	18.6 (1.2)	18.8 (1.3)***	19.0 (1.2)	18.3 (1.8)	18.7 (1.5)***
BMI ≥ 18.5, *n* (%)	0	0	0	22 (71.0%)	17 (53.1%)	39 (61.9%)***	19 (67.9%)	11 (40.7%)	30 (54.5%)***

Eating disorder psychopathology									
Overall severity (global EDE)	3.02 (1.3)	–	3.02 (1.3)	1.35 (1.1)	–	1.35 (1.1)***	1.49 (1.3)	–	1.49 (1.3)***
Overall severity (global EDE-Q)	3.61 (1.5)	2.74 (1.4)*	3.19 (1.5)	1.45 (1.23)	1.15 (1.4)	1.30 (1.3)***	1.68 (1.3)	1.23 (1.5)	1.46 (1.4)***
Global EDE-Q < 1 SD above the community mean,^a^*n* (%)	8 (25.8%)	16 (50.0%)	24 (38.1%)	28 (90.3%)	27 (87.1%)	55 (88.7%)***	22 (78.6%)	21 (80.8%)	43 (79.6%)***
Dietary restraint (EDE-Q subscale)	3.63 (1.9)	2.91 (1.8)	3.32 (1.8)	1.31 (1.4)	0.86 (1.3)	1.09 (1.4)***	1.62 (1.6)	1.14 (1.7)	1.39 (1.7)***
Eating concern (EDE-Q subscale)	3.46 (1.4)	2.71 (1.6)	3.09 (1.6)	1.17 (1.2)	0.97 (1.2)	1.07 (1.2)***	1.32 (1.4)	1.55 (1.7)	1.43 (1.5)***
Shape concern (EDE-Q subscale)	3.90 (1.7)	2.86 (1.6)*	3.40 (1.7)	2.02 (1.6)	1.65 (1.8)	1.83 (1.7)***	1.61 (1.6)	1.10 (1.5)*	1.36 (1.6)***
Weight concern (EDE-Q subscale)	3.46 (1.8)	2.46 (1.5)*	2.95 (1.7)	1.31 (1.2)	1.13 (1.5)	1.22 (1.4)***	2.17 (1.4)	1.14 (1.4)**	1.67 (1.5)***

Eating disorder behaviour (EDE-Q)									
Binge eating, *n* (%) present	12 (40.0%)	5 (15.6%)*	17 (27.4%)	8 (26.7%)	2 (6.3%)*	10 (16.1%)	7 (25.0%)	3 (11.5%)	10 (18.5%)
If present, episodes/28 days, median (range)	2.5 (1–20)	4.0 (2–40)	3.0 (1–40)	5.0 (1–10)	5.0 (2–8)	5.0 (1–10)	3 (1–8)	28 (25–28)*	5.0 (1–28)
Self-induced vomiting, *n* (%) present	4 (12.9%)	9 (28.1%)	13 (20.6%)	1 (3.2%)	1 (3.1%)	2 (3.2%)**	2 (7.1%)	5 (19.2%)	7 (13.0%)
If present, episodes/28 days, median (range)	9 (1–45)	15 (1–40)	14 (1–45)	11.0	30.0	20.5 (11–30)	3 (2–4)	10 (1–25)	4 (1–25)
Laxative misuse, *n* (%) present	2 (6.5%)	3 (9.4%)	5 (7.9%)	1 (3.2%)	0 (0%)	1 (1.6%)	2 (7.1%)	3 (11.5%)	5 (9.3%)
If present, episodes/28 days, median (range)	8 (2–14)	8 (4–15)	8 (2–15)	1.0	–	1.0	10.5 (1–20)	4.0 (3–28)	4.0 (1–28)

General psychiatric features, GSI	1.54 (0.8)	1.37 (0.7)	1.45 (0.8)	0.68 (0.6)	0.67 (0.6)	0.67 (0.6)***	0.83 (0.7)	0.56 (0.5)	0.70 (0.6)***

EDE – Eating Disorder Examination (version 16.0D) (Fairburn , Cooper, & O'Connor 2008). EDE-Q – Eating Disorder Examination Questionnaire (version 6.0) (Fairburn & Beglin, 2008). GSI – Global Severity Index (Derogatis, 1977; Derogatis & Spencer, 1982). *p < 0.05; **p < 0.01 UK vs Italian patients and total sample vs baseline.

a Global EDE-Q less than 1SD above community EDE-Q mean for young adult women (Mond et al., 2006) (i.e., <2.77).
